# Harnessing Nitrogen-Fixing and Phosphate-Mobilizing Bacteria for Sustainable Agriculture

**DOI:** 10.3390/microorganisms14040803

**Published:** 2026-04-01

**Authors:** Madina Rakhmatova, Tokhir Khusanov, Khabibjon Kushiev, Zhanar Tekebayeva, Zuobin Wang, Aliya Temirbekova, Ainur Amantayeva, Akhan Abzhalelov, Zhandarbek Bekshin, Arvind Kumar Dubey, Fariza Kyzykbaikyzy, Arman Abilkhadirov, Aslan Temirkhanov, Zhadyrassyn Nurbekova

**Affiliations:** 1 Scientific Research Institute of Agrobiotechnology and Biochemistry, Gulistan State University, Gulistan 120100, Uzbekistan; madinaraxmatova110895@gmail.com (M.R.); kushiev@mail.ru (K.K.); 2 The Institute of Microbiology of Academy of Sciences of Uzbekistan, Tashkent 100128, Uzbekistan; tokhir.khusanov1985@gmail.com; 3 Republican Collection of Microorganisms, Astana 010000, Kazakhstan; zanartekebaeva@gmail.com (Z.T.); atemirbekova94@gmail.com (A.T.); ainuramantaeva91@gmail.com (A.A.); ab_akhan@mail.ru (A.A.); zmbekshin@gmail.com (Z.B.); good_alien@mail.ru (A.A.); 4 International Research Centre for Nano Handling and Manufacturing of China, Changchun University of Science and Technology, Changchun 130012, China; wangz@cust.edu.cn; 5 Department of Agronomy and Horticulture, University of Nebraska Lincoln, Lincoln, NE 68588, USA; arvindbiotech28@gmail.com; 6 Department of Biotechnology and Microbiology, L.N. Gumilyov Eurasian National University, Astana 010000, Kazakhstan; farizakyzykbaj@gmail.com

**Keywords:** plant growth-promoting rhizobacteria, biofertilizers, biotic stress, abiotic stress, stress tolerance

## Abstract

This review investigates the multifaceted roles of nitrogen-fixing and phosphate-mobilizing bacteria in natural ecosystems, with a particular focus on their contributions to plant growth and sustainable soil management. These microbial communities contribute substantially to nutrient cycling by converting atmospheric nitrogen into plant-available forms and mobilizing insoluble phosphorus in soil, thereby enhancing soil fertility and promoting sustainable plant productivity. This review synthesizes current knowledge on the mechanisms underlying biological nitrogen fixation, phosphate solubilization and mineralization, and the production of plant growth–promoting metabolites. Particular attention is given to plant–microbe interactions and their role in improving nutrient availability, regulating plant physiological processes, and enhancing tolerance to abiotic stresses such as salinity, drought, and heavy metal contamination. The findings underscore the ecological importance of these plant-associated microbial communities and highlight their potential applications in biofertilizer and biostimulant development for sustainable agriculture and reduced dependence on synthetic fertilizers.

## 1. Introduction

Currently, there are nearly 8 billion people worldwide, and this number is expected to increase to almost 9 billion by 2050 [1]. The intensive use of chemical fertilizers over the past decades has significantly increased global food production. However, several studies have highlighted inefficiencies and regional disparities in fertilizer use, which have contributed to environmental pollution, soil nutrient imbalances, and unsustainable agricultural practices. The excessive application of inorganic fertilizers and pesticides in agriculture can lead to declining soil fertility and environmental degradation.

Soil is considered a limited and non-renewable natural resource [2]. Increasing soil degradation, including salinization and declining soil fertility, has become a major challenge for sustainable agriculture [3,4]. In this context, the use of beneficial microorganisms as biofertilizers has emerged as an environmentally friendly strategy to improve crop productivity while maintaining soil health. These microorganisms can enhance soil fertility, stimulate plant growth, and improve plant tolerance to abiotic and biotic stresses, thereby contributing to sustainable food production.

**Figure 1:**
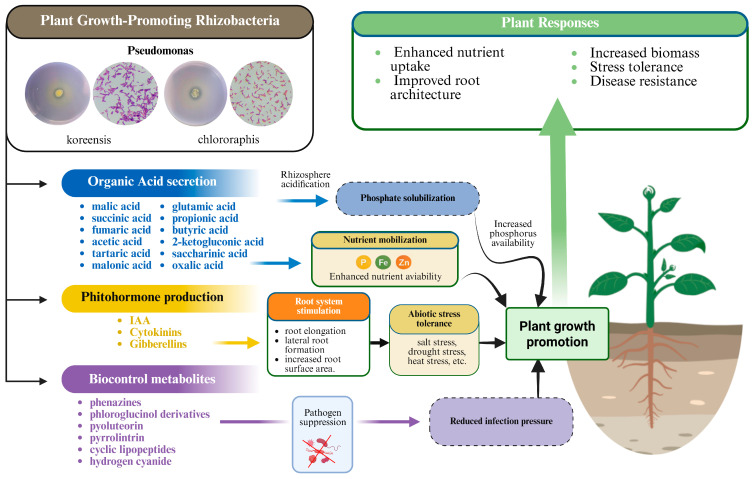
Integrated mechanisms of plant growth promotion mediated by plant growth–promoting rhizobacteria [13,14,15,16].

One of the most promising approaches involves the use of plant growth–promoting rhizobacteria (PGPR). These beneficial microorganisms are known to promote plant growth and development by enhancing nutrient availability, suppressing phytopathogens, and even stimulating plant defense systems against biotic and abiotic stress conditions (Figure 1) [5,6]. Among them, nitrogen-fixing bacteria (NFB) play a crucial role by converting atmospheric nitrogen (N_2_) into biologically available forms for plant uptake. Nitrogen is an essential macronutrient required for plant growth and development, as it is a structural component of amino acids, nucleic acids, proteins, and chlorophyll molecules [7]. However, atmospheric nitrogen cannot be directly utilized by plants and must first undergo biological nitrogen fixation. Biological nitrogen fixation is mediated by NFB, known as diazotrophs, and is catalyzed by the enzyme complex nitrogenase. Because nitrogenase is highly sensitive to oxygen, many diazotrophic microorganisms perform nitrogen fixation under anaerobic or microaerobic conditions to prevent enzyme inactivation [8,9].

Another important group of beneficial microorganisms includes phosphate-mobilizing bacteria (PMB), which increase the availability of phosphorus in the rhizosphere and facilitate its uptake by plant roots. Phosphorus is the second most important macronutrient for plants after nitrogen and plays a key role in metabolic processes such as photosynthesis, energy transfer, and nucleic acid synthesis [10]. Certain soil bacteria can convert these insoluble phosphates into bioavailable orthophosphate forms through the secretion of organic acids and phosphatase enzymes, thereby enhancing phosphorus availability in the soil [11]. Representative phosphate-mobilizing bacteria include *Pseudomonas*, *Enterobacter*, *Agrobacterium*, and *Bacillus* species [12]. Because nitrogen and phosphorus are often present in soil in forms unavailable to plants, microbial processes that convert these nutrients into bioavailable forms are critical for plant nutrition and soil fertility. Therefore, harnessing nitrogen-fixing and phosphate-mobilizing bacteria offers a promising strategy for improving agricultural productivity while reducing dependence on synthetic fertilizers. However, despite significant progress in understanding plant growth–promoting microorganisms, the mechanisms, ecological roles, and agricultural applications of nitrogen-fixing and phosphate-mobilizing bacteria remain scattered across the literature and lack a comprehensive synthesis in the context of sustainable agriculture and plant stress tolerance. This review aims to summarize the current knowledge on nitrogen-fixing and phosphate-mobilizing bacteria and their roles in sustainable agriculture. Specifically, the review focuses on the mechanisms of biological nitrogen fixation and phosphate mobilization, the ecological roles of these microorganisms in plant–microbe interactions, and their potential applications as biofertilizers for improving crop productivity under abiotic and biotic stress conditions.

## 2. Role of Plant Growth–Promoting Microorganisms in Enhancing Plant Tolerance to Stresses

Plant growth–promoting rhizobacteria (PGPR) play an important role in enhancing plant tolerance to environmental stresses through multiple biochemical and physiological mechanisms. These microorganisms improve plant health by facilitating nutrient acquisition, regulating phytohormone levels, and activating plant defense responses. Consequently, PGPR-mediated stress tolerance represents an important ecological function that supports plant productivity under adverse environmental conditions.

Plant PGPR can enhance plant tolerance to both abiotic and biotic stresses. There is abundant evidence showing the significant role that microorganisms play in plant growth and development [17]. Several soil microorganisms have been identified that possess the ability to promote plant growth. These microorganisms enhance plant productivity by facilitating nutrient acquisition, participating in nutrient cycling, fixing nitrogen, producing phytohormones, and increasing tolerance to abiotic and biotic stresses [18]. Certain bacterial RNases protect plants from viral diseases. In such cases, a promising method of plant protection is the use of biopreparations based on microorganisms that produce RNases [19]. Enzyme production by bacterial strains also contributes to soil nutrient cycling and organic matter degradation. The strains *Bacillus licheniformis*, *Bacillus subtilis*, and *Bacillus halotolerans* have demonstrated activity in producing protease, amylase, and cellulase enzymes during cellular metabolism, highlighting their role in the degradation of organic matter and nutrient cycling [20].

### 2.1. Role of Plant Growth–Promoting Microorganisms in Enhancing Plant Tolerance to Abiotic and Biotic Stresses

The plant develops in close association with complex microbial communities, especially microorganisms known as the phytomicrobiome [21]. Microorganism communities have co-existed with terrestrial plants since their earliest evolutionary stages, helping them overcome challenges such as limited nutrient availability, exposure to new and harsh environmental conditions, and attacks from different pathogens [22]. The rhizosphere is a narrow zone surrounding plant roots and is therefore tightly associated with the microorganisms inhabiting the soil and the root secretion [23]. The rhizosphere hosts a rich and diverse microbial community. It includes bacteria, algae, protozoa, fungi, and actinomycetes; among them, bacteria represent the most abundant group [24]. As in all microenvironments, not only do microorganisms interact with each other, but they also have a significant impact on their host, which in this case is the plant root system. These microbes provide numerous beneficial and essential functions to the plant, such as nutrient acquisition and plant growth promotion, while the plant, in turn, supplies them with carbon-containing compounds and root exudates that serve as an energy source [25,26]. Rhizobacteria, or root-associated bacteria, are a common group in the rhizosphere. A subgroup of rhizobacteria that exerts direct or indirect beneficial effects on plant growth and development is known as plant growth–promoting rhizobacteria (PGPR) [17]. PGPR have multiple mechanisms of action, which may vary depending on plant species, environmental conditions, and microbial interactions. These mechanisms include nitrogen fixation, mineral solubilization and mineralization, phytohormone production, and siderophore secretion, thereby improving nutrient uptake and plant development [27,28]. Backer et al. (2018) reported that PGPR establish close associations with plant roots in the rhizosphere and play a vital role in improving plant tolerance to abiotic stresses such as salinity, drought, and heat, as well as biotic stresses caused by pathogens [29]. The adverse impact on plants, referred to as stress, can be classified into two major groups: abiotic and biotic stresses. These stresses include salinity, cold, heavy metals, UV-C irradiation, and pathogen attacks by bacteria, fungi, and viruses. Such stresses increase the production of reactive oxygen species (ROS), which generate reactive carbonyl species leading to plant senescence, crop losses, and global declines in agricultural productivity [30,31]. Under such stress conditions, plant growth–promoting bacteria (PGPB) influence plant development through both direct and indirect mechanisms. Direct mechanisms include the production of phytohormones, nitrogen fixation, modulation of water relations, and phosphate mobilization [32]. Several phytohormones, including indole-3-acetic acid (IAA), gibberellic acid (GA_3_), zeatin, abscisic acid (ABA), and ethylene, can be regulated by PGPB [32]. PGPR can improve plant water relations by enhancing root development and water uptake under drought and salinity stress conditions. Indirectly, PGPB produce antibiotics, hydrogen cyanide, and volatile organic compounds (VOCs) to combat biotic stresses such as pathogen invasion [33].

Direct mechanisms are usually involved in response to abiotic stress conditions. One of the main phytohormones associated with stress tolerance mediated by PGPB is ethylene. It plays a key role in plant responses to stress conditions and is often referred to as a stress hormone. High levels of ethylene can lead to senescence of various tissues, chlorophyll, and the abscission of leaves, flowers, and petals [34]. Many PGPBs produce ACC deaminase, which directly inhibits the production of ethylene precursor ACC and reduces stress-induced ethylene accumulation [35]. Another phytohormone strongly associated with stress response is abscisic acid, which plays a pivotal role among other phytohormones during stress conditions, regulating key processes during stress such as stomatal closure, senescence, fruit ripening, and seed dormancy [36]. Several studies have shown that PGPR inoculation can modulate ABA-related stress signaling pathways, thereby improving plant tolerance to drought and salinity conditions [37,38].

In addition to phytohormone regulation, PGPB can produce osmoprotectants such as proline and trehalose, which are crucial for adaptation to salinity and other osmotic stresses [37]. Osmoprotectants play an essential role in maintaining cellular homeostasis, including protein structure stability, redox balance, and relative water content [39]. These compounds contribute to improved plant tolerance to osmotic stress conditions. Several rhizosphere bacterial strains have been reported to produce phytohormones and osmolytes that enhance plant stress tolerance [40].

On the other hand, under biotic stress, PGPB use indirect defense mechanisms. Plants exposed to biotic stress activate defense pathways such as systemic acquired resistance (SAR) and induced systemic resistance (ISR) [41]. Specific signaling molecules trigger these pathways through molecular signaling cascades that coordinate plant defense responses against pathogens [42]. SAR is typically associated with the accumulation of salicylic acid and pathogenesis-related proteins such as chitinase and glucanase. In contrast, ISR, which is often activated by PGPR such as *Pseudomonas* spp., involves signaling pathways regulated by jasmonate and ethylene [43]. The simultaneous activation of ISR and SAR enhances plant defense across a wider range of pathogens compared to the action of either pathway alone [44]. Rhizobacteria with resistance-inducing and antagonistic properties can be utilized to develop novel inoculants that combine different mechanisms of action by enhancing the effectiveness of biocontrol strategies and promoting more sustainable crop production systems [45,46]. Various microbial genera, including *Pseudomonas*, *Bacillus*, *Burkholderia*, *Trichoderma*, and *Streptomyces*, suppress plant pathogens through antibiotic production, enzyme secretion, and volatile compounds that activate plant defense responses. In the study of Ryu et al. (2004), VOCs such as 2,3-butanediol emitted by *Bacillus subtilis* GB03 and *Bacillus amyloliquefaciens* IN937a activated systemic resistance in *Arabidopsis thaliana*, reducing disease severity caused by *Erwinia carotovora* [47]. In addition, siderophore production by antagonistic bacteria limits pathogen growth through iron chelation while simultaneously promoting plant development by enhancing iron uptake [48].

Table 1 summarizes recent findings on various plant growth-promoting microorganisms (PGPMs) and their roles in controlling abiotic and biotic stresses across different crops. Collectively, these studies demonstrate that plant–microbe interactions, particularly those involving PGPR, significantly enhance plant tolerance and growth performance even under adverse environmental conditions. Such beneficial microorganisms represent sustainable and eco-friendly alternatives to chemical pesticides and fertilizers in modern agriculture.

PGPRs contribute to plant health and productivity through multiple mechanisms, including nutrient mobilization, production of phytohormones, and enzymatic activities that regulate plant growth and development. They also strengthen plant immune responses against pathogens, thereby reducing disease susceptibility and minimizing the reliance on chemical inputs (Figure 1). The application of one or more microbial species to plants lacking such beneficial communities has proven effective in improving nutrient uptake, enhancing tolerance to abiotic stresses, and increasing overall productivity [49]. Furthermore, understanding the diversity among antagonistic microbial populations that share similar biocontrol traits is essential for optimizing biocontrol strategies [48]. PGPRs can also function as biocontrol agents through biopriming—a technique involving the inoculation of seeds with beneficial microorganisms to protect them from seed- and soil-borne diseases [50]. This method promotes uniform germination and vigorous seedling growth in many horticultural crops, offering an effective, sustainable, and environmentally safe alternative to chemical treatments.

**Table 1:** The role of PGPB in enhancing the tolerance of inoculated plants under abiotic and biotic stress conditions.

PGPB Strain (Examples)	Crop	Stress Factor	Main Plant Response	Significance	Experimental Conditions	Key Limitations	References
*Brachybacterium saurashtrense*, *brevibacterium casei*, *haererohalobacter* spp.	*Arachis hypogaea*	Salinity	Increased root/shoot biomass and overall growth	Demonstrates improved salt tolerance mediated by rhizosphere bacteria	Laboratory-based study: hydroponic culture	Must be tested under field conditions, crop specificity, duration and growth stage (seedlings), and salinity specificity	[51]
*Hartmannibacter diazotrophicus*, *pseudomonas* sp.	*Medicago sativa*	Salinity	Enhanced nodulation, chlorophyll content, and photosynthetic rate	Demonstrates microbial support of nitrogen fixation and physiological stability under saline conditions	Laboratory-based study: growth room	Must be tested under field conditions, limited genetic diversity, no long-term impact data/	[52]
*Lactobacillus* sp. *and P. putida* *Azotobacter chroococcum*	*Lactuca sativa* and *Raphanus sativus seeds*	Salinity	Raised the plumule and radicle length of germinated seeds	Demonstrates microbial priming of plant physiological response	Laboratory-based study: growth room	Must be tested under field conditions, limited genetic diversity, no long-term impact data/	[52]
*Bacillus cereus* pb25	*Vigna radiata*	Salinity	Increased antioxidant enzyme activity and osmolyte accumulation	Demonstrates mitigation of oxidative stress induced by salinity	Laboratory-based study: pot experiment	Sterilized soil environment, limited area for root growth, specific scope of bacteria	[53]
*Bacillus subtilis*, *pseudomonas fluorescens*	*Solanum lycopersium*	Bacterial and fungal pathogens	Reduced disease incidence and pathogen growth	Demonstrates rhizobacterial biocontrol against soil-borne pathogens	Laboratory-based studies: pot experiment	Pathogen titer dependency, environmental sensitivity	[54,55,56]
*Burkholderia phytofirmans*, *Pseudomonas fluorescens*	*Arabidopsis thaliana*, *Solanum lycopersium*	Pathogen infection	Activation of induced systemic resistance (ISR) and defense enzymes	Demonstrates microbial priming of plant immune responses	Laboratory-based studies	Inconsistent pathogen protection, plant genotype specificity, sensitivity to inoculation methods	[57,58,59,60,61]
*Streptomyces* spp., *Bacillus velezensis*	*Solanum lycopersium*, *Oriza sativa*	Fungal pathogens	Suppressed pathogen development and improved plant growth	Demonstrates actinobacterial and Bacillus-based biological disease control potential	Laboratory-based studies: pot experiments	Focused on single variety, molecular complexity, sterile soil environment, lack of field validation	[62,63,64]
*Trichoderma harzianum*	*Solanum lycopersium*, *Cucumis sativus*	Soil-borne pathogens	Reduced root damage and increased seedling vigor	Demonstrates combined growth promotion and disease suppression	Laboratory-based studies: greenhouse	Strain-dependent colonization variability	[56,65,66]
*Pseudomonas libanesis* TR1, *Pseudomonas reactans* Ph3R3	*Brassica oxyrrhina*	Drought	Increased plant growth, leaf relative water content and pigment levels; reduced proline and malondialdehyde accumulation	Demonstrates improved drought tolerance through regulation of plant water status and oxidative stress	Laboratory-based studies: growth chamber	Single crop species validation	[67]
*Sinorhizobium medicae*	*Medicago truncatula*	Drought	Enhanced root nodulation and nutrient acquisition	Demonstrates the importance of rhizobial symbiosis for maintaining nutrient uptake under water deficit	Laboratory-based studies: greenhouse	Legume-specific symbiotic dependency	[68]

### 2.2. Role of NFB and PMB in Plant Drought Stress Tolerance

Nitrogen-fixing bacteria (NFB) and phosphate-mobilizing bacteria (PMB) play a pivotal role in enhancing plant resilience to drought stress through diverse physiological and biochemical mechanisms [5,69]. Beneficial microbes that accumulate around the rhizosphere assist plant growth and development through direct and indirect mechanisms. Indirect mechanisms include the production of phytohormones by bacteria, which help plants survive under stress conditions. For example, to highlight the effect of phytohormones on exopolysaccharide secretion and ACC deaminase activity [70,71]. These mechanisms enhance plant adaptation to drought stress by improving root architecture, water retention, and stress signaling pathways. For instance, PGPR-produced indole-3-acetic acid (IAA) promotes root elongation and lateral root formation, thereby enhancing water uptake under drought conditions (Table 2) [72]. Abscisic acid (ABA) also plays a central role in drought response by regulating stomatal closure and maintaining cellular water balance [73]. Microbial osmolytes work synergistically with plant-produced osmolytes to maintain plant health. PGPR inoculation has been shown to enhance the accumulation of osmolytes, such as glycine betaine, in plants under drought stress. For example, Gou et al. found that maize treated with *Klebsiella variicola* F2, *Raoultella planticola* YL2, and *Pseudomonas fluorescens* YX2 accumulated more glycine betaine and choline than the control, resulting in improved water relations and plant growth under drought conditions (Table 2). The accumulation of osmolytes such as glycine betaine (GB) protects plants from abiotic stress through osmoregulation or osmoprotection [39]. In addition, microbial ACC deaminase activity reduces stress-induced ethylene levels, thereby promoting root growth and improving plant tolerance to drought conditions [74,75]. Through direct interactions with plant roots, PGPR further enhance drought tolerance by improving plant physiological stability.

For example, the association of *Pseudomonas putida* and *Bacillus thuringiensis* reduces stomatal conductivity and electrolyte leakage in buds and roots due to the accumulation of proline [76,77].

A growing body of research indicates that nitrogen-fixing bacteria (NFB) and phosphate-mobilizing bacteria (PMB) contribute to improved plant adaptation to water-deficit conditions, although the extent of plant response varies depending on plant species, microbial strain compatibility, and environmental conditions. A substantial proportion of available evidence originates from experiments performed under controlled laboratory or greenhouse settings, which may not fully represent the complexity of natural field environments characterized by fluctuating soil moisture regimes, temperature variability, and dynamic interactions with indigenous microbial communities. These factors may influence microbial survival, colonization efficiency, and functional stability, thereby contributing to inconsistent performance of microbial inoculants under field conditions [4,78].

**Table 2:** Drought stress tolerance mechanisms in plants mediated by NFB and PMB.

Target Crop	Microorganism(s)	Specific Biochemical Mechanism	Molecular Targets	Engineering Potential	Experimental Conditions	Limitations	Refs.
*Zea mays*	*Azospirillum lipoferum*	Increased accumulation of soluble sugar, free amino acids, and proline	Upregulation of *P5CS* genes for proline biosynthesis	Metabolic engineering for enhanced osmoprotectant production	Greenhouse-based pot experiment. Conducted under controlled conditions by using plastic pots	Sterile soil environment, needs open field conditions, limited genetic scope	[79]
*Zea mays*	*Bacillus* spp.	Reduced electrolyte leakage and decreased antioxidant enzyme activity (CAT, GPX)	SOD and CAT gene expression regulation	Engineering strains with superior ROS-scavenging capabilities	Glasshouse pot experiment	Lack of complex field variabilities, failure to measure grain yield, unknown molecular signaling mechanisms	[76]
*Triticum aestivum*	*Azospirillum brasilense* NO40	Bacterial-mediated attenuation of specific transcript levels	Modulation of *ERF* (Ethylene Response Factor) transcription factors	Precision gene silencing of drought-induced senescence	Laboratory-based experiment	Focused solely on the seedling stage, inability to account for field-level soil and climate complexity, narrow evaluation on specific bacterial strains and cultivars	[80]
*Triticum aestivum*	*Rhizobium leguminosarum* (LR-30)	Production of Catalase, Exopolysaccharides (EPS), and IAA. Enhanced chlorophyll and ascorbic acid; lower browning intensity. Decreased stomatal conductance and MDA; increased relative leaf water content	*iaaM/H* (auxin) and *exo/wge* (EPS) gene clusters	Enhancement of biofilm stability in arid soil microenvironments	Laboratory-based experiment: sterilized glass jars	Sterile soil environment, lack of field validation, focused on seedling stage only, artificial drought stress	[81]
*Medicago sativa* L.	*Sinorhizobium medicae*	Sustained root nodulation and nutrient acquisition during water deficit	*nod* and *nif* gene expression stability	Developing drought-resilient nitrogen-fixing symbioses	Greenhouse experiment, utilized pot-grown plants	Potential pot-restricted root growth, use of a model organism rather than crop plants, lack of complex, multi-stress environmental factors	[68]

### 2.3. Role of NFB and PMB in Plant Salinity Stress Tolerance

Under salinity stress conditions, nitrogen-fixing bacteria (NFB) and phosphate-mobilizing bacteria (PMB) contribute significantly to plant adaptation by modulating key physiological and biochemical processes [5,6]. Soil salinization is a widespread environmental constraint that significantly limits plant growth and agricultural productivity worldwide [3]. This is one of the most significant stress factors negatively affecting plant growth and development. Salinity leads to a decrease in the soil water potential, making it difficult for plants to absorb water and nutrients from the soil, which results in osmotic stress. The nitrogen fixation process is one of the most important processes under saline stress conditions. Plant growth–promoting microorganisms, including NFB and PMB, have been shown to alleviate the adverse effects of salinity stress in various crops (Table 3) [82]. For example, rice roots of KDML105 treated with *Streptomyces venezuelae* ATCC 10712, which produces ACC deaminase, demonstrated better salt tolerance compared to untreated rice [83]. Similarly, *Pseudomonas stutzeri* has been reported to reduce the negative impact of salinity in both salt-tolerant and salt-sensitive chili plants. *Azospirillum*-treated lettuce seeds showed better germination and vegetative growth compared to the control under salinity conditions [84]. These beneficial effects are mediated through mechanisms such as phytohormone regulation, osmotic adjustment, and antioxidant defense. PGPR improves salt stress by accumulating compounds that maintain cellular osmotic balance and protect plant tissues under saline conditions (Table 3) [76]. (*Bacillus pumilus* and *Bacillus subtilis* isolated from saline soils showed positive traits such as producing IAA, ammonia, and hydrogen cyanide (HCN), phosphate solubility, and salt stress tolerance [85]. *Acinetobacter* spp. and *Pseudomonas* sp. produce ACC deaminase and IAA in barley and maize under salt stress, promoting plant growth [86].

*Pseudomonas pseudoalcaligenes* and *Bacillus pumilus* reduce lipid peroxidation and superoxide dismutase activity in salt-sensitive GJ-17 under salt stress [87].

Extensive studies have highlighted the important role of NFB and PMB in mitigating the adverse effects of salt stress on plant growth and productivity, yet the magnitude of beneficial outcomes depends on salinity intensity, plant genotype, microbial strain specificity, and soil physicochemical characteristics. Much of the current knowledge is derived from studies conducted in controlled experimental systems, where environmental variability is minimized, and therefore may not adequately capture the heterogeneity of field conditions characterized by uneven salt distribution, changing moisture regimes, and ecological interactions with native soil microbiota. These environmental and ecological factors may influence microbial colonization efficiency, functional gene expression, and long-term persistence in the rhizosphere, leading to variability in plant growth–promoting performance under field conditions [34,78].

**Table 3:** Salinity  stress tolerance mechanisms in plants mediated by NFB and PMB.

Target Crops	Microorganisms	Key Biological Mechanism	Experimental Conditions	Key Limitations	References
*Vigna radiata, Hordeum vulgare* L.	*Rhizobium*, *H. diazotrophicu*	ACC deaminase activity: reduction in ethylene-induced growth stunting.	Laboratory-based study: axenic conditions using growth pouches and jars	Lack of competition from native soil microorganisms, using only early growth stages, limited salinity range	[88]
*Triticum aestivum*, *Lactuca sativa*	*Azospirillum* sp.	Accumulation of compatible solutes (proline, soluble sugars) and iron sequestration.	Controlled pot experiment conducted in a wire house	Lack of natural environmental fluctuations, minimal microbial competition compared to natural ecosystems	[89]
*Oriza sativa (GJ-17)*	*P. pseudoalcaligenes*, *P. pumilus*	Scavenging of ROS: regulation of antioxidant enzymes	Greenhouse-based pot experiment	Limited duration, lack of field variability, focused on single, salt-sensitive cultivar	[87]
*Oriza sativa*	*B. amyloliquefaciens* (SN13)	Global modulation of stress-responsive transcription factors (14+ specific genes)	Laboratory and greenhouse experiment	Focus on early seedling growth only, lack of field environmental variables, and reliance on specific salt concentration	[90]

### 2.4. Role of NFB and PMB in Plant Heavy Metal Stress Tolerance

Under heavy metal stress conditions, nitrogen-fixing bacteria (NFB) and phosphate-mobilizing bacteria (PMB) contribute to plant adaptation primarily through detoxification, transformation, and immobilization mechanisms that reduce metal toxicity and improve plant growth [91,92]. Anthropogenic activities, including industrialization and intensive agriculture, have led to the accumulation of heavy metals in soils beyond permissible limits, posing a serious threat to plant growth and soil health. Heavy metals are metallic elements with a density greater than 4 g/cm^3^, and cannot be degraded biologically, and are toxic even at low concentrations [67,93]. Conventional remediation methods are often costly and may negatively affect soil structure, highlighting the need for sustainable alternatives. In this context, phytoremediation, supported by plant–microbe interactions, has emerged as an environmentally friendly approach for mitigating heavy metal contamination [67].

Some microorganisms are capable of transforming or immobilizing heavy metals through various biochemical processes, thereby reducing their toxicity and bioavailability in the environment. For example, plant growth-promoting rhizobacteria like *Pseudomonas* sp. participate in the biotransformation of Fe (III), Zn, and Cd-citrate complexes and influence their mobility and bioavailability [94].

One of the key mechanisms involved is the regulation of metal availability through biochemical interactions in the rhizosphere. Siderophores are low molecular weight organic compounds produced by PGPR that chelate heavy metals and influence their distribution in the soil–plant system. In addition to their role in iron acquisition, siderophores contribute to metal binding and transport processes within the rhizosphere [95,96]. The metabolites and organic acids produced by PGPR play an effective role in phytoremediation. Gluconic, oxalic, and citric acids are considered the most effective in modifying metal solubility and bioavailability, thereby facilitating either mobilization or stabilization depending on environmental conditions [97,98]. Furthermore, biomethylation represents an additional transformation mechanism, involving the transfer of methyl groups through bacterial activity. Many bacteria participate in the methylation of Pb, Hg, Se, As, and Sn [99]. Heavy metal-resistant bacterial genera such as *Bacillus*, *Lysinibacillus*, and *Pseudomonas* spp. enhance phytoremediation efficiency through multiple functional traits, including metal tolerance and plant growth promotion [100]. In addition, microbial production of metal-binding peptides such as phytochelatins contributes to intracellular sequestration and detoxification of heavy metals, further supporting plant survival under contaminated conditions [101].

Increasing attention has been directed toward the use of NFB- and PMB-associated microorganisms as supportive agents for enhancing plant tolerance to heavy metal toxicity and improving phytoremediation efficiency, although their effectiveness is strongly influenced by soil characteristics, metal speciation, contamination levels, and plant–microbe compatibility. Most experimental findings are based on simplified laboratory or greenhouse investigations that do not fully replicate the complex physicochemical dynamics of contaminated field soils, where variations in pH, organic matter content, competing ions, and indigenous microbial populations can significantly influence microbial survival and metal transformation processes. Consequently, these factors may contribute to inconsistent phytoremediation performance under field conditions [34,102].

## 3. Types of Nitrogen-Fixing Microorganisms and Mechanisms of Biological Nitrogen Fixation

Nitrogen-fixing bacteria (NFB), also known as diazotrophs, establish diverse ecological associations with plants, particularly through symbiotic interactions in the root tissues of legumes (Fabaceae). The most common legumes include dry beans, chickpeas (*Cicer arietinum* L.), cowpeas (*Vigna unguiculata* L.), lentils (*Lens esculenta* L.), and peanuts (*Arachis hypogaea* L.). These plants form symbiotic associations with NFB, enabling efficient biological nitrogen fixation and contributing to improved soil fertility in intercropping and crop rotation systems [102].

**Figure 2:**
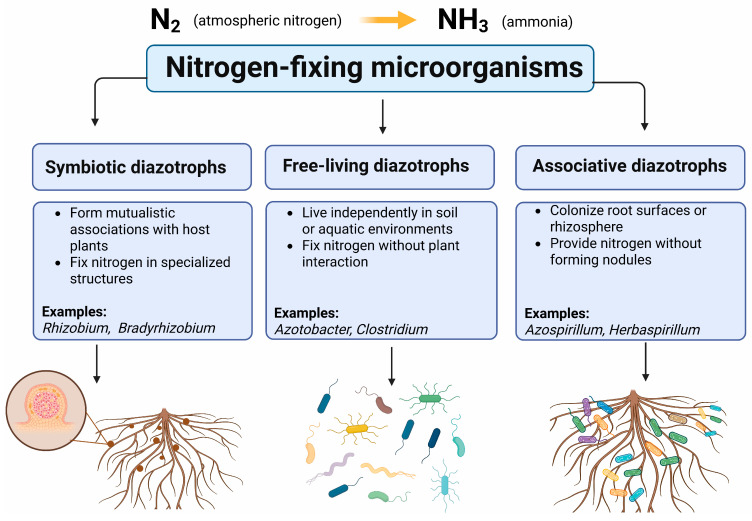
Ecological classification of nitrogen-fixing microorganisms.

Free-living diazotrophs such as *Azotobacter chroococcum* and *Azotobacter vinelandii* are commonly found in tropical soils, whereas *Azotobacter beijerinckii* is more frequently associated with acidic environments [103]. Nitrogen-fixing microorganisms (diazotrophs) are phylogenetically diverse and occur in a wide range of ecological habitats. Genomic and molecular studies have demonstrated that diazotrophs are distributed across multiple bacterial and archaeal lineages and are present in various environments, including soils, plant-associated habitats, and aquatic ecosystems [104,105]. Nitrogen-fixing bacteria can be broadly classified into three ecological groups according to their association with plants: symbiotic diazotrophs, free-living diazotrophs, and associative diazotrophs (Figure 2).

Several symbiotic NFB, including *Allorhizobium*, *Azoarcus*, *Azorhizobium*, *Bradyrhizobium*, *Burkholderia*, *Frankia*, *Mesorhizobium*, *Rhizobium*, and *Sinorhizobium,* have been identified [9]. Examples of free-living diazotrophs include *Azoarcus*, *Azospirillum*, *Azotobacter*, *Gluconacetobacter*, and *Herbaspirillum* [102]. Free-living diazotrophs represent a relatively small component of the plant rhizosphere ecosystem and can be classified into several phylogenetic groups, including:

Alpha-proteobacteria (*Rhizobia, Bradyrhizobia, Rhodobacteria*)

• Beta-proteobacteria (Burkholderia, Nitrosospira).
• Gamma-proteobacteria.
• Cyanobacteria [28].

With respect to oxygen sensitivity, Clostridium pasteurianum, a strict (obligate) anaerobe, is considered an important nitrogen-fixing bacterium under anaerobic conditions. In contrast, facultative anaerobes such as Klebsiella oxytoca can also fix nitrogen, but this process generally occurs only when oxygen is absent or present at very low levels in the system [106]. Some obligate aerobic diazotrophs, such as Azotobacter vinelandii, have evolved physiological mechanisms to protect nitrogenase from oxygen. These bacteria maintain high respiratory activity through cytochrome oxidases, rapidly consuming oxygen and thereby creating intracellular conditions that allow nitrogen fixation to proceed [97,106].

In addition, endophytic NFB inhabit internal plant tissues. Several studies suggest that the internal plant microbiome may provide advantages compared with external microbial communities, such as those found in the rhizosphere or phyllosphere. Endophytic bacteria may experience reduced competition and predation and can interact more directly with plant metabolic processes, allowing even low concentrations of microbial metabolites to influence plant physiology [98,107].

Endophytic bacteria often exhibit a degree of host specificity, meaning that certain strains are closely associated with particular plant species or varieties. As a result, biostimulant formulations based on these microorganisms may demonstrate optimal performance in the plant hosts from which they were originally isolated. However, many biofertilizer formulations are successfully developed using endophytic or rhizospheric bacteria and can exhibit broad-spectrum plant growth–promoting effects across different crops. Nevertheless, the efficiency of colonization and plant growth–promoting activity may vary among plant species. Therefore, careful selection and evaluation of microbial strains are essential to ensure the effectiveness and wider applicability of biostimulant products in agricultural systems. Biological nitrogen fixation is the process by which atmospheric nitrogen (N_2_) is converted into ammonia (NH_3_), a form that can be assimilated by plants. In symbiotic systems, rhizobacteria induce structural and physiological changes in plant roots, leading to the formation of specialized structures known as nodules, where nitrogen fixation occurs. In addition to these symbiotic associations, many NFB exist as free-living microorganisms that are widely distributed in agricultural soils. These free-living diazotrophs represent an important natural source of nitrogen in non-symbiotic nitrogen fixation (SNF) within both natural and agricultural ecosystems [103].

Under microaerobic conditions, atmospheric nitrogen is reduced to ammonia as follows: N_2_ + 8H^+^ + 8e^−^ + 16Mg-ATP → 2NH_3_ + H_2_ + 16Mg-ADP + 16Pi

Nitrogen fixation is catalyzed by nitrogenase enzymes, whose activity depends on a complex metal cofactor known as the FeMo-cofactor (FeMoco). This enzyme complex facilitates the reduction of atmospheric nitrogen into ammonia, which can subsequently be incorporated into plant metabolic pathways [13].

Despite significant advances in understanding nitrogenase function, two main pathways have been proposed: the “distal” and the “alternative” pathways [108,109]. In the distal pathway, hydrogenation occurs first at the terminal nitrogen atom, followed by reduction in the metal-bound nitrogen. In contrast, the alternative pathway involves alternating hydrogenation between the two nitrogen atoms until ammonia is released [108,110]. These pathways are distinguished by their intermediate species, including nitrido intermediates (distal pathway) and diazene or hydrazine intermediates (alternative pathway) [111,112]. Although direct observation of intermediates in native nitrogenase remains challenging, studies using model complexes have provided indirect evidence supporting both pathways [111]. Investigations involving molybdenum generally support the distal pathway, whereas iron-based systems tend to support the alternative pathway [111,112]. Experimental studies using molybdenum-based model systems support the distal pathway, whereas iron-based clusters and vanadium nitrogenase systems are more consistent with the alternative mechanism [111,113]. However, the absence of clearly defined intermediates in the native enzyme indicates that the exact reaction mechanism remains unresolved, and computational studies continue to support both pathways depending on the proposed active site within the MoFe cofactor [108,111].

## 4. Beneficial Bacteria in the Plant Root Microbiome and Phyllosphere

Building on the mechanisms of nitrogen fixation discussed above, plant-associated microbial communities extend beyond root symbioses to complex microbiomes that inhabit both below- and above-ground plant compartments. Diverse bacterial communities colonize both the root tissues of legumes and the rhizosphere of non-leguminous plants, collectively forming the plant root microbiome. The root microbiome (the microbial communities present in plant roots and the rhizosphere) plays a central role in regulating plant growth, nutrient acquisition, and stress resilience [75]. Microbial communities are of significant importance in food production systems due to their potential benefits in improving plant health and protecting crops from pathogen attacks [5]. Microbiome manipulation strategies include the application of exogenous beneficial microorganisms and the enrichment of native microbial communities. Direct inoculation with antagonistic or plant growth–promoting microorganisms is widely used to enhance crop-associated microbiomes [72].

Plant-associated microbiomes are spatially structured and include endophytic, epiphytic, and rhizospheric communities, each contributing differently to plant physiology and ecosystem functioning [49].

A common type of epiphytic phytomicrobiome is the phyllosphere. The phyllosphere includes the surface of leaf tissues and the apoplast [114]. NFB detected in the phyllosphere of crops such as wheat, cotton, and maize includes genera such as *Beijerinckia*, *Azotobacter*, and *Derxia* [115,116]. Phyllosphere bacteria that promote plant growth and prevent diseases produce bioactive metabolites and enzymes that can enhance plant health as well as help mitigate damage to leaves caused by harmful airborne substances, pesticide residues, or plastics [115,117].

In addition to epiphytic colonization, some microorganisms originate from root or seed endophytes and migrate through the vascular system to aerial plant parts, contributing to phyllosphere microbial diversity. Generally, bacteria are more commonly found on the lower surfaces of leaves than on the upper surfaces [114].

The global surface area of the phyllosphere is more than 4 × 10^8^ km^2^ [118]. Nitrogen fixation rates in the phyllosphere vary significantly depending on environmental conditions and plant species. In some tropical habitats, nitrogen fixation levels in tree phyllospheres have been recorded at over 60 kg N ha^−1^, whereas in temperate systems the rates are considerably lower [116].

Evidence from multiple studies confirms the presence of diazotrophic communities in the phyllosphere of various crop plants [117]. Nitrogen fixation predominantly occurs on leaf surfaces rather than within internal tissues, and cyanobacteria associated with epiphytes are considered major contributors to this process. Additionally, diazotrophic γ-proteobacteria and related taxa may also participate in phyllosphere nitrogen fixation [119,120]. Recent advances in high-throughput sequencing and metagenomic approaches have significantly improved the understanding of phyllosphere microbial communities and their functional potential. Metagenomic analyses of leaf-associated microbiomes have revealed a diverse assemblage of diazotrophic microorganisms and nitrogen-metabolism genes in the phyllosphere [121]. Similarly, DNA metabarcoding studies targeting nifH genes have confirmed the presence of diverse nitrogen-fixing bacterial taxa associated with leaf surfaces [122]. Comprehensive reviews of the phyllosphere microbiome also emphasize the ecological importance of leaf-associated microbial communities in plant nutrient cycling and ecosystem functioning [123].

## 5. Types and Significance of Phosphate-Mobilizing Bacteria and Mechanisms of Phosphate Mobilization

In addition to nitrogen fixation, phosphorus availability represents a major limiting factor for plant productivity, and phosphate-mobilizing bacteria (PMB) play a critical role in enhancing phosphorus bioavailability in soil systems [11]. In agricultural soils, a large proportion of applied phosphorus fertilizers becomes rapidly immobilized through interactions with soil minerals, limiting its availability for plant uptake. The synthesis of chemical phosphorus fertilizers requires large amounts of high-quality phosphate rock, which significantly contributes to the depletion of global phosphorus reserves. Microbial-based strategies, particularly those involving PMB, have emerged as sustainable alternatives to improve phosphorus use efficiency and reduce dependence on chemical fertilizers. Phosphate mobilization includes multiple processes, among which phosphate solubilization represents an important mechanism by which plant-associated bacteria convert insoluble phosphorus into plant-available orthophosphate. Findings have shown that many endophytic strains produce gluconic acid (GA) in concentrations ranging from 14 to 169 mM and exhibit moderate to high phosphate-solubilizing capacities (~400–1300 mg L^−1^). Application of such strains to phosphate-limited systems has been shown to significantly enhance plant growth and nutrient acquisition [124]. Phosphorus is an essential macronutrient required for plant growth and development, playing a central role in energy transfer, nucleic acid synthesis, and membrane structure. In soils, phosphorus occurs in both inorganic mineral forms and organic compounds; however, the majority exists in insoluble forms that are not readily available to plants [125,126]. The discrepancy between plant phosphorus demand and soil availability makes phosphorus deficiency a major constraint in agricultural systems [125]. Although phosphate fertilizers are commonly applied to overcome this limitation, their efficiency is low due to rapid fixation in soil. Soluble phosphorus reacts with aluminum and iron minerals, forming insoluble complexes. As a result, less than 30% of applied phosphorus is typically available for plant uptake [127]. Additionally, a significant portion is lost through runoff and leaching, leaving only 10–20% available for plant uptake [128]. Moreover, phosphate fertilizers depend on non-renewable phosphate rock reserves, which are projected to become increasingly limited [129]. According to several studies, various bacterial species, particularly those colonizing the rhizosphere, possess the ability to solubilize organic phosphates or dissolve insoluble inorganic phosphate compounds such as tricalcium phosphate, dicalcium phosphate, hydroxyapatite, and rock phosphate [130]. Application PMB has been shown to reduce fertilizer requirements while maintaining crop productivity [6,131]. In addition, PMB contribute to soil remediation processes, including heavy metal stabilization and nutrient cycling [124,132]. Phosphate-mobilizing bacteria also exhibit multifunctional traits, including the production of organic acids, siderophores, and phytohormones, which contribute not only to phosphorus solubilization but also to improved nutrient cycling and stress tolerance. In addition, some PMBs can facilitate the mobilization of metal complexes, highlighting their potential role in soil remediation processes [132]. Numerous bacterial strains have demonstrated phosphate-solubilizing activity under laboratory conditions; however, their performance under field conditions remains variable and requires further investigation [6,133,134].

Mechanisms of phosphate mobilization. Phosphorus (P) is one of the most limiting nutrients for plants, with the productivity of approximately 30–40% of the world’s arable land constrained by its availability [135,136]. During soil formation and aging (pedogenesis), P transitions from a mineral form to a labile form (soluble orthophosphate, PO_4_^3−^) that is available for plant uptake. However, much of the labile P is adsorbed onto mineral surfaces, immobilized in the organic matter of the soil, or incorporated into non-available inorganic forms. These processes result in reduced phosphorus bioavailability and accumulation of recalcitrant phosphorus pools [137,138]. Organic phosphorus, particularly phytate, can account for 20–95% of total soil phosphorus [139,140]. Phytate exhibits strong binding and immobilization in soils compared to orthophosphate [140]. Due to its high negative charge, phytate strongly adsorbs onto soil components [141], forming stable Fe/Al–phytate complexes on mineral and organic surfaces [142]. Microbial solubilization of inorganic phosphate represents a key mechanism by which plant-associated bacteria enhance plant growth.

These microorganisms release organic acids and chelating compounds that dissolve phosphate complexes and convert them into bioavailable orthophosphate [143]. Soil microorganisms facilitate phosphorus cycling by mobilizing both inorganic and organic phosphorus pools through metabolic activity, thereby converting inaccessible phosphorus forms into plant-available forms.

Microbial phosphate mobilization occurs through three primary mechanisms:

1. Solubilization of inorganic phosphate via soil acidification and secretion of organic acids and metal-chelating compounds [144,145].
2. Mineralization of organic phosphorus through extracellular enzymes such as phosphatases, phytases, and C–P lyases [144,146].
3. Transformation of Fe- and Al-bound phosphorus complexes into bioavailable forms [129,147].

To date, research has predominantly focused on inorganic phosphorus availability, while organic phosphorus transformation remains comparatively less explored [144,145]. However, soil pH and mineralogy are key regulators of phosphorus solubility, and should be considered when evaluating microbial phosphate solubilization under field conditions. Fe-bound organic phosphorus (e.g., phytate complexes) represents a major limitation to phosphorus cycling and availability in soils. The release of bioavailable phosphorus is mediated by microbial enzymes such as phytases, phosphonatases, and C–P lyases [15]. Among these mechanisms, organic acid production and acid phosphatase activity are considered dominant processes in phosphate solubilization [148]. Gluconic acid is one of the most effective organic acids involved in inorganic phosphate solubilization [149,150]. Several studies have reported that in Gram-negative bacteria, the direct oxidation pathway of glucose (DOPG) leads to the production of glucose dehydrogenase (GCD) and gluconate dehydrogenase (GAD) [151]. These enzymes function in the periplasmic space, enabling substrate oxidation and organic acid production. Consequently, organic acids are released into the surrounding environment, lowering pH and promoting the dissolution of mineral phosphates [125]. Phosphate-solubilizing bacteria produce a wide range of organic acids, including gluconic, citric, oxalic, lactic, and acetic acids, which contribute to phosphate solubilization through acidification and metal chelation [152]. The type and quantity of organic acids produced depend on microbial metabolism and available carbon sources [153]. Organic acid production reduces soil pH, thereby enhancing phosphate solubilization efficiency, particularly through periplasmic oxidation pathways in Gram-negative bacteria [15]. The production of organic acids leads to a decrease in environmental pH. A lower pH reflects the secretion of organic acids by phosphate-solubilizing microorganisms through direct oxidation pathways occurring at the outer side of the cytoplasmic membrane [152].

## 6. Application of Nitrogen-Fixing and Phosphate-Mobilizing Bacteria as Biostimulants

Building on the mechanisms of nutrient mobilization and stress tolerance discussed in previous sections, microbial biostimulants represent a key application of nitrogen-fixing bacteria (NFB) and phosphate-mobilizing bacteria (PMB) in sustainable agriculture. A plant biostimulant is any substance or microorganism applied to plants, regardless of its nutritional content, aimed at improving nutrient efficiency, abiotic stress tolerance, and crop quality. The term also encompasses commercial formulations containing these biologically active components [154].

Microbial biostimulants, particularly NFB and PMB, enhance plant growth by improving nutrient availability through biological nitrogen fixation and phosphate solubilization in the rhizosphere. Plant growth–promoting rhizobacteria (PGPR) play a central role in these processes by mobilizing nutrients, producing phytohormones, and enhancing plant resilience under stress conditions.As a result, they offer a sustainable alternative to excessive reliance on chemical fertilizers and pesticides. The most commonly used bacterial genera as biostimulants include *Azospirillum*, *Acetobacter*, *Azotobacter*, and *Pseudomonas*. In addition, *Pseudomonas* and *Bacillus* species are widely recognized for their dual roles as plant growth promoters and biocontrol agents. These microorganisms enhance nutrient availability, improve competitive interactions in the rhizosphere, and induce systemic resistance mechanisms in plants, thereby protecting plants from pathogens and improving overall plant health. Microbial biostimulants are increasingly applied in agricultural systems worldwide, highlighting their potential as multifunctional inputs that support sustainable crop production while reducing environmental impacts associated with chemical inputs [18]. Phosphate-mobilizing bacteria (PMB) primarily contribute to improved phosphorus availability, enhanced nutrient use efficiency, and increased plant tolerance to abiotic stress conditions [155]. Plant biostimulants do not act as direct nutrient sources or pesticides; instead, they stimulate plant physiological processes that enhance nutrient uptake and stress tolerance. Biostimulants can be classified based on their composition, mode of action, or both [154,156,157]. A wide range of bacteria and fungi have been investigated for their biostimulant potential, with rhizosphere-associated microorganisms such as *Rhizobium* and PGPR representing key functional groups. Numerous commercial microbial biostimulant products have been developed based on PGPR and related microorganisms, demonstrating their practical applicability in modern agriculture [25].

Despite their considerable potential, the effectiveness of microbial biostimulants under field conditions often varies depending on environmental factors, soil characteristics, formulation stability, and microbial survival after application. Differences in inoculation methods, carrier materials, and compatibility between introduced strains and native microbial communities may influence the consistency of plant growth–promoting effects across different agroecosystems. Therefore, further optimization of formulation technologies, strain selection, and multi-location field validation studies is required to improve the reliability and large-scale adoption of NFB- and PMB-based biostimulants [78].

**Figure 3:**
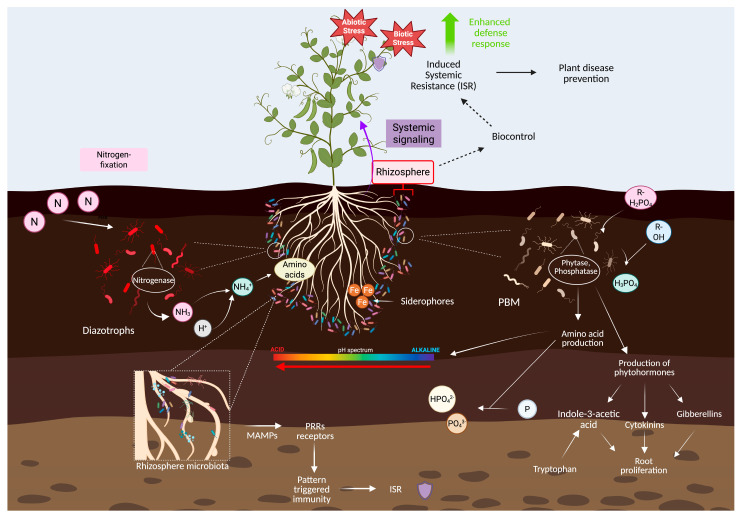
Integrated conceptual framework of plant–microbe–soil interactions in sustainable agriculture. The schematic illustrates the roles of plant growth-promoting rhizobacteria (PGPR), including biological nitrogen fixation, phosphate solubilization, siderophore production, phytohormone synthesis, and induction of systemic resistance (ISR). These interconnected processes enhance nutrient availability, improve plant stress tolerance under abiotic and biotic conditions, and support the development and application of microbial biostimulants for sustainable crop production.

To integrate the diverse microbial mechanisms discussed in this review and their application as biostimulants, a conceptual framework summarizing plant–microbe–soil interactions in sustainable agriculture is presented (Figure 3). This framework highlights the coordinated roles of nitrogen-fixing and phosphate-mobilizing microorganisms, phytohormone production, nutrient cycling, and induced systemic resistance in enhancing plant growth and resilience under stress conditions.

## 7. Challenges in Field Application and Commercialization of Nitrogen-Fixing and Phosphate-Mobilizing Bacteria

Despite significant progress in identifying and describing NFB and PMB as plant growth-promoting agents, their translation from lab-scale research to field-level agricultural technologies remains limited. Multiple interconnected ecological, technological, regulatory, and adoption barriers collectively limit the consistency and scalability of microbial biofertilizer applications. Backer et al. (2018) reported that this variability is largely driven by environmental fluctuations and interactions with native soil microbiota [29]. Similarly, Timmusk et al. (2017) identified poor reproducibility in field applications as a key bottleneck for large-scale use [6].

### 7.1. Variability of Field-Level Performance

One of the major challenges limiting the large-scale application of NFB and PMB is the inconsistency of their performance under natural field conditions. Even though many bacterial strains demonstrate strong functional efficiency under controlled laboratory environments, their agronomic effectiveness frequently declines under field conditions. This distinction reflects the influence of fluctuating environmental parameters, such as temperature, moisture, soil pH, nutrient status, and seasonal dynamics, which collectively regulate microbial adaptation, metabolic activity, and persistence in the rhizosphere [28]. This instability during field application reflects the complex interactions between introduced inoculants and indigenous soil microbiota, which often compete for ecological niches, carbon substrates, and root colonization sites, rather than being influenced solely by abiotic environmental stress [25]. Therefore, even metabolically efficient strains may fail to establish stable rhizosphere populations capable of sustaining NFB or PMB activity over time. This ecological competition is a major determinant of the frequent variability in plant growth–promoting effects across agroecosystems [6]. Furthermore, increasing evidence suggests that the functional gene expression in beneficial microorganisms is influenced by environmental context, resulting in reduced expression of nitrogenase activity and organic acid–mediated phosphate solubilization pathways under stressful soil conditions [15]. Together, these findings show that the primary limitations related to field-level variability are not the intrinsic metabolic potential of beneficial strains, but rather the difficulty of maintaining their functional stability within heterogeneous soil ecosystems.

### 7.2. Rhizosphere Ecological Complexity and Microbial Interactions

The rhizosphere is one of the most dynamic and biologically complex ecological niches in terrestrial environments, characterized by intense microbial competition, cooperation, signaling interactions, and resource partitioning processes [25]. Within this complex microenvironment, the establishment and persistence of introduced NFB and PMB depend not only on their physiological capabilities but also on their compatibility with existing microbial communities. New strains frequently encounter antagonistic microorganisms that suppress colonization efficiency through antibiotic production, niche exclusion, or competition for root exudate–derived carbon sources. Simultaneously, beneficial synergistic interactions with native microbiota are not always predictable, further contributing to variability in functional outcomes following inoculation [29]. Thus, microbial inoculant performance is strongly context-dependent and influenced by site-specific ecological factors. Recent advances in rhizosphere microbial ecology suggest that plant growth promotion is rarely mediated by single microbial species acting independently, but rather emerges from complex microbial consortia operating within structured ecological networks [25]. This perspective highlights the limitations of traditional pure-strain inoculation strategies and emphasizes the need for next-generation biostimulant formulations that leverage functionally complementary microbial communities to maintain ecological resilience under fluctuating environmental conditions.

### 7.3. Formulation Stability and Shelf-Life Constraints

Another major limitation affecting the commercialization potential of NFB and PMB is the development of stable and reliable formulation technologies that preserve microbial viability during storage, transportation, and field application. While lab-scale cultivation allows precise control of environmental conditions that support microbial growth, maintaining high cell viability and functional activity during industrial-scale production remains more challenging [29]. Many microbial inoculants show a progressive reduction in cell density, metabolic activity, and stress tolerance during storage, particularly when exposed to temperature fluctuations, desiccation, and oxidative damage. These factors influence shelf life and reduce the reliability of field performance following application. As a result, formulation stability represents a critical technological bottleneck limiting the widespread adoption of microbial biostimulants [6]. Additionally, carrier material selection also plays a decisive role in determining microbial survival and colonization efficiency after soil application. Differences in carrier composition, moisture retention capacity, nutrient availability, and protective properties significantly affect inoculant persistence within the rhizosphere environment [29]. The absence of universally optimized carrier systems further contributes to variability in product performance across different agricultural settings.

### 7.4. Industrial Scale-Up and Quality Control Limitations

The transition from lab-scale cultivation to industrial-scale production introduces additional technical limitations that directly affect the feasibility of commercialization. Large-scale fermentation processes require optimization of oxygen transfer efficiency, agitation conditions, and contamination control strategies in order to maintain consistent biomass yield and functional activity [29]. Even minor deviations in these parameters may significantly affect inoculant quality and reproducibility. Equally important is the lack of standardized quality control protocols governing the production of microbial biostimulants. Variability in cell density, strain purity, and contamination levels can lead to inconsistent product performance under field conditions, thereby reducing market acceptance [33]. The establishment of harmonized quality assessment frameworks, therefore, is essential for improving reliability and ensuring reproducibility across commercial inoculant formulations.

Moreover, inconsistencies associated with formulation technologies and delivery systems further complicate the translation of well-characterized laboratory strains into reliable commercial products. These technological limitations highlight the need for integrated production strategies that combine optimized fermentation processes with advanced formulation engineering approaches to preserve microbial functionality throughout the product life cycle [29].

### 7.5. Regulatory Barriers, Field Validation Gaps, and Farmer Adoption Constraints

Additionally, regulatory and socio-economic factors influence the commercialization pathway of microbial biostimulants. In many regions, the absence of harmonized regulatory frameworks governing microbial inoculant registration, quality standards, and performance validation delays product approval and restricts market accessibility [29]. Regulatory uncertainty also limits private-sector investment in large-scale production infrastructure and formulation innovation. Moreover, multi-location field validation trials are required to demonstrate consistent agronomic benefits across diverse environmental conditions. Although numerous studies report promising laboratory-scale results, comparatively fewer investigations provide robust field-scale performance datasets necessary for large-scale technology deployment [6,15]. This lab-to-field translation gap remains one of the most critical barriers preventing the widespread adoption of nitrogen-fixing and phosphate-mobilizing bacteria in modern agricultural systems.

Finally, farmer adoption is influenced by perceptions of reliability, cost-effectiveness, and compatibility with existing crop management practices. Compared with conventional chemical fertilizers, microbial biofertilizers are often perceived as less predictable in their performance, which reduces their attractiveness in risk-sensitive agricultural environments [29]. Improving farmer awareness through demonstration trials, extension programs, and evidence-based performance validation will therefore play a decisive role in accelerating the transition toward biologically based nutrient management strategies.

## 8. Conclusions and Future Perspective

Due to the increasing impact of abiotic, biotic, and anthropogenic stresses, sustainable crop production has become increasingly challenging. Rapid population growth and climate variability continue to intensify global food security challenges. The excessive reliance on synthetic fertilizers has contributed to soil degradation, nutrient imbalance, and environmental pollution. In this context, improving plant–microorganism–soil interactions has emerged as a critical strategy for sustainable agriculture. This review highlights that nitrogen-fixing bacteria (NFB) and phosphate-mobilizing bacteria (PMB) play central and complementary roles in enhancing plant growth, nutrient use efficiency, and stress tolerance. Through diverse physiological and biochemical mechanisms, including nutrient mobilization, phytohormone production, and stress regulation, these microorganisms significantly contribute to plant resilience under adverse environmental conditions. Importantly, the application of these functional microorganisms as biostimulants provides a promising pathway toward reducing dependence on chemical inputs while maintaining crop productivity and environmental sustainability. Despite significant advances in understanding the mechanisms and ecological roles of NFB and PMB, their interactions within complex soil–plant–microbiome systems remain insufficiently understood. In particular, the integration of nutrient cycling, stress tolerance, and microbial community dynamics requires further investigation to fully exploit their potential in agricultural systems. Key knowledge gaps include limited field-scale validation of microbial inoculants, insufficient understanding of microbial interactions under diverse environmental conditions, and a lack of integration of multi-omics approaches (e.g., metagenomics, transcriptomics) to elucidate functional mechanisms in situ. Future research should focus on the development of stable and efficient microbial consortia, the application of systems biology approaches to optimize plant–microbe interactions, and the integration of microbial biostimulants into precision agriculture frameworks. Additionally, exploring climate-resilient microbial strains will be essential to address the challenges posed by global climate change and environmental variability. Particular attention should also be given to improving formulation technologies, enhancing shelf-life stability, and ensuring functional consistency of microbial products across different agroecological environments. However, several challenges must be addressed for large-scale implementation, including variability in field performance, limited shelf-life and formulation stability of microbial products, and regulatory constraints associated with biofertilizer commercialization. Addressing these technological, ecological, and regulatory limitations will be essential to facilitate the reliable adoption of microbial biostimulants in modern agricultural practices. Overall, harnessing the functional potential of NFB and PMB represents a sustainable and environmentally sound strategy for future agriculture; however, translating laboratory findings into reliable field applications will be the key determinant of their long-term success.

## Data Availability

All the data are available in the text.
